# Effects of balance training distribution on balance performance in adolescents

**DOI:** 10.1055/a-2898-7328

**Published:** 2026-07-29

**Authors:** Anna Maria Wissmann, Mohamed El Houssaini, Mathew W. Hill, Thomas Muehlbauer

**Affiliations:** 1 Division of Movement and Training Sciences/Biomechanics of Sport 27170 Universität Duisburg-EssenEssenNorth-Rhine-WestfaliaGermany; 2 Division of Movement and Training Sciences/Biomechanics of Sport 27170 University of Duisburg-EssenEssenNRWGermany; 3 School of Psychology and Vision Sciences 4488 University of LeicesterLeicesterEnglandUnited Kingdom of Great Britain and Northern Ireland; 4 Division of Movement and Training Sciences/Biomechanics of Sport 27170 Universitat Duisburg-EssenEssenGermany

**Keywords:** postural control, practice, treatment, Adolescence, dose-response relationship, adaption

## Abstract

It is firmly established that balance training leads to significant improvements in postural control in youth. However, little is known about how differences in balance training distribution (i.e., how total training volume is scheduled: duration×frequency), despite the equal total training volume, affect training-induced adaptations. This study was aimed at determining the effect of balance training distribution on balance performance.

Twenty-five adolescents (15.8±0.6 y) were randomly assigned to either a 4-week balance training–3×/wk group (massed practice: 4 weeks of balance training for three sessions/wk) or a 6-week balance training–2×/wk group (distributed practice: 6 weeks of BT for two sessions/wk) group. Balance assessments included the timed One-Legged Stance test and the Y Balance Test Lower–Quarter.

Significant pretest to post-test improvements were detected for all parameters of the One-Legged Stance test and Y Balance Test Lower–Quarter, irrespective of balance training distribution. Additionally, a group×test interaction ( *p* =0.041 and *η*_p_^2^ =0.17) was detected for the posterolateral reach distance in favor of the massed practice group.

These findings highlight that most outcomes improved regardless of balance training distribution, and only one outcome showed a benefit for the combination of shorter balance training duration with higher balance training frequency (i.e., massed practice) than vice versa (i.e., distributed practice).

## Introduction

Previous research showed that balance training (BT) improves balance performance in adolescents 1 2 3 . Schedler et al 2 reported improvements in the timed One-Legged Stance (OLS) test and greater reach distances in the Y Balance Test Lower–Quarter (YBT–LQ) after 7 weeks of BT in adolescents. Although these findings highlight the benefits of BT, it remains unclear which BT distribution (i.e., how the total training volume is scheduled) yields the greatest improvements. A previous meta-analysis 1 revealed that BT durations typically ranged from 4 to 6 weeks, and BT frequencies from two to three sessions/wk. However, to date, no study has directly compared different BT distributions while keeping the total training volume equal. For practitioners, a short BT duration with high BT frequency (i.e., massed practice) represents an efficient approach, as balance improvements may be achieved within a short period. However, from a theoretical perspective, it has been argued that distributed practice (i.e., long BT duration combined with a low BT frequency) is superior for adaptations 4 5 . This advantage arises from deeper cognitive processing 6 , enhanced consolidation 7 , and more effective memory retrieval 8 . A study involving young adults showed that the acquisition of a new gait pattern benefited more from distributed practice 9 . However, there is a lack of studies involving adolescents, and it is not possible to directly apply previous findings due to immaturities in postural control mechanisms at this age 10 11 .

The present study compared the effectiveness of BT programs with equal total volume but differences in distribution–either massed (4 weeks, 3×/wk) or distributed (6 weeks, 2×/wk) practice–on balance outcomes in adolescents. We hypothesized that both BT programs would lead to improvements in balance 1 2 3 , but distributed practice would elicit greater gains due to more time for adaptation processes 4 6 8 9.

## Materials and methods

### Participants

An a priori power analysis ( *f* =0.25, *α* =0.05, 1 − *β* =0.80, *r* =0.70, groups=2, and assessments=2) revealed that a total sample size of *N* =22 participants (i.e., *n* =11 per group) would be sufficient to detect significant within–between interaction effects 12 . Thus, 25 male adolescents were randomly assigned to either a 4-week BT–3×/wk group ( *n* =12, age: 15.8±0.6 y, stature: 177.5±4.4 cm, mass: 68.7±9.4 kg, body mass index [BMI]: 21.8±2.6 kg/m ^2^ , and sports club participation: 9.0±2.1 y) or a 6-week BT–2×/wk group ( *n* =13, age: 15.9±0.6 y, stature: 174.2±5.9 cm, mass: 72.4±10.5 kg, BMI: 23.8±2.9 kg/m ^2^ , and sports club participation: 8.2±2.0 y). There were no differences in participant characteristics between groups. Group allocation was performed using a coin flip by the first author. The second author conducted the balance assessments and was blinded to group allocation. All participants were healthy (i.e., no known musculoskeletal or neurological impairments) and gave their written consent to participate. Additionally, written consent was obtained from the legal guardians of minors before the study began. The study was approved by the local Human Ethics Committee.

### Balance assessments

**Fig. 1:**
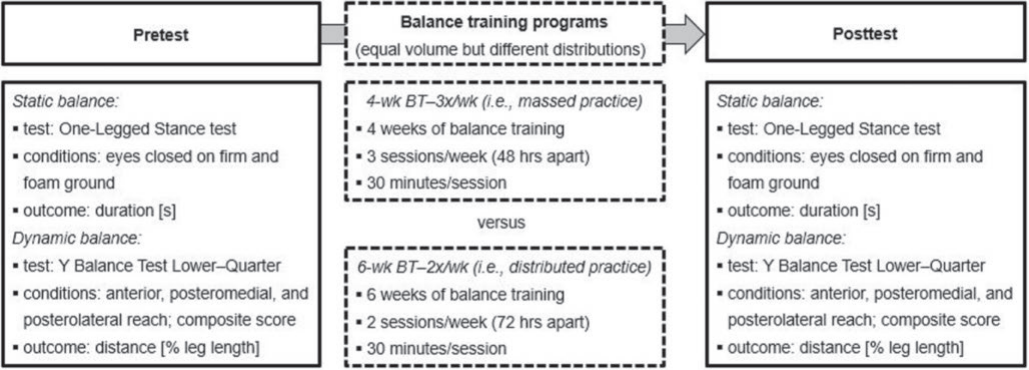
Schematic description of the study design.

Assessments were conducted in a gymnasium by a qualified sport scientist before and after each training period ( Fig. 1 ). Static balance was assessed using the timed OLS test. Participants stood barefoot on their non-dominant leg for, as long as possible, up to a maximum of 60 seconds. The OLS test was performed under two conditions: (1) eyes closed on the firm ground (EC–FI) and (2) eyes closed on a foam surface (AIREX balance pad; EC–FO). After one practice trial, one measurement trial was conducted, and the maximum stance time (seconds) recorded for each condition was used for analysis.

Dynamic balance was measured using the YBT–LQ. Participants stood barefoot with their non-dominant leg positioned on the central footplate and reached as far as possible with their dominant leg in the anterior (AT), posteromedial, and posterolateral (PL) directions. After three practice trials, three measurement trials were completed, and the absolute maximal reach distance (cm) for each direction was recorded. These values were then normalized by dividing the absolute reach distance by leg length (LL) and multiplying by 100. Normalized composite score (CS; % LL) was calculated by summing the maximal reach distances across all three directions, dividing by three times LL, and multiplying by 100. Limb length was measured from the AT superior iliac spine to the most distal point of the medial malleolus 13 . Validity and reliability of both tests have established 14 15 16 17 18 .

### Balance training programs

In line with published recommendations 1 19 , the same standardized BT program was conducted for four (three sessions/wk, 48 h apart, 30 min each) and six (two sessions/wk, 72 h apart, 30 min each) weeks, respectively. Each session included five balance exercises including unipedal (1) stance on a balance pad, (2) on a wobble board, (3) weight shifting, (4) vertical jump-landings, and (5) horizontal jump-landings that were performed twice per leg. Progression was achieved by switching from practice with open to closed eyes and changing exercise duration from 20 to 45 s or repetitions from 10 to 15. Thus, the total BT volume was equal between groups and increased from 7 minites pre session to 15 minutes per session.

### Statistical analysis

Descriptive data are reported as group means with 95% confidence intervals. For all analyses, assumptions of normality (Shapiro–Wilk test) and homogeneity of variance/sphericity (Mauchly test) were checked and met prior to conducting parametric analyses. A 2 (group: 4-wk BT–3×/wk, 6-wk BT–2×/wk)×2 (test: pretest, posttest) repeated measures analysis of variance (ANOVA) was conducted to detect training-related group differences. For significant interactions, Bonferroni-corrected post hoc tests ( *t* -test) were employed. Using partial eta-squared ( *η*_p_^2^ ), effects of the ANOVA were estimated as being either small (0.02≤ *η*_p_^2^ ≤0.12), medium (0.13≤ *η*_p_^2^ ≤0.25), or large ( *η*_p_^2^ ≥0.26), whereas Cohen’s *d* was used to classify the effects of post hoc tests as being either trivial (0≤ *d* ≤0.19), small (0.20≤ *d* ≤0.49), medium (0.50≤ *d* ≤0.79), or large ( *d* ≥0.80) 20 . The level of significance was set at *p* < 0.05 and all analyses were performed using SPSS version 27.0.

## Results

There were no significant differences in pretest values between groups. For static balance, ANOVA showed significant large-sized main effects of test for both stance conditions ( Table 1 ). This indicates improvements in stance duration independent of the applied BT distribution. The main effect of group and the group×test interaction were not significant. For dynamic balance, ANOVA showed significant large-sized main effects of test for all reach direction and the CS ( Table 1 ). Further, a significant group×test interaction was found for the PL reach direction. Post hoc tests revealed significant improvements from pretest to posttest in both groups (4-wk BT–3×/wk: *t* =–3.048, *p* =0.006, *d* =0.60 and 6-wk BT–2×/wk: *t* =–2.628, *p* =0.011, *d* =0.10), with the effect size being medium for the 4-week BT-3×/wk group but trivial for the 6-week BT–2×/wk group. The main effect of group was not significant.

**Table:** **Table 1** Effects of BT distribution on measures of static and dynamic balance in male adolescents

Meaure	4-wk BT–3×/wk ( *n* =12)		6-wk BT–2×/wk ( *n* =13)		*p* -Value ( *η*_p_^2^ )		
	Pretest	Post-test	Pretest	Post=test	Group	Test	Group *x* Test
OLS							
OLS time; EC-FI (s)	38.3 (25.5–51.1)	50.8 (42.4–59.1)	42.7 (30.1–55.3)	53.8 (47.6–59.9)	0.520 (0.02)	**0.003 (0.32)**	0.854 (0.00)
OLS time; EC-FO (s)	10.1 (6.1–14.1)	18.7 (12.4–24.9)	6.8 (2.6–10.9)	13.7 (7.5–19.9)	0.204 (0.07)	**<0.001 (0.61)**	0.526 (0.02)
YBT-LQ							
YBT-LQ: AT reach (% LL)	76.8 (73.2–80.4)	80.7 (77.7–83.7)	77.9 (73.5–82.3)	83.6 (78.6–88.6)	0.341 (0.03)	**<0.001 (0.42)**	0.447 (0.03)
YBT-LQ: PM reach (% LL)	114.3 (109.4–119.2)	120.6 (116.1–125.1)	117.1 (111.6–122.6)	121.7 (115.6–127.8)	0.566 (0.02)	**<0.001 (0.57)**	0.403 (0.03)
YBT-LQ: PL reach (% LL)	111.4 (107.6–115.2)	116.3 (110.8–121.8)	116.2 (107.8–124.5)	117.5 (109.4–125.7)	0.497 (0.02)	**<0.001 (0.39)**	**0.041 (0.17)**
YBT-LQ: CS (% LL)	100.8 (97.1–104.5)	105.9 (101.9–109.8)	103.7 (98.2–109.2)	107.6 (102.0–113.2)	0.459 (0.02)	**<0.001 (0.71)**	0.349 (0.04)

Abbreviations: AT, anterior; BT, balance training; CS, composite score; EC, eyes closed; FI, firm ground; FO, foam ground; LL, leg length; OLS, One-Legged Stance test; PL, posterolateral; PM, posteromedial; wk, week; YBT-LQ, Lower Quarter Y Balance test.

Note: Values are group means with 95% confidence intervals in brackets and *p* -values with effect sizes ( *η*_p_^2^ ) in brackets. 0.02≤ *η*_p_^2^ ≤0.12 indicates small, 0.13≤ *η*_p_^2^ ≤0.25 indicates medium, and *η*_p_^2^ ≥0.26 indicates large effects. Bold values indicate statistically significant differences.

## Discussion

Consistent with the first part of our hypothesis and previous research 2 3 both groups showed improvements in static and dynamic balance outcomes. In contrast to the second part of our hypothesis and findings from previous studies in adults 4 8 9 , most outcomes improved regardless of BT distribution and only one outcome (i.e., PL reach distance) showed a larger gain in favor of the massed practice group. Given the same total training volume, these findings indicate that massed and distributed practice – resulting in 48 versus 72 hours between consecutive sessions – provides sufficient time for training-related ongoing recovery, adaptation, and consolidation processes 6 7 21 . This interpretation is consistent with the findings of Paillard 21 , who reported the recovery of the postural control system from fatigue after ≈20 minutes, as well as Taubert et al 22 who found changes in brain areas responsible for postural control after 1 hour of BT. Both BT distributions provided sufficient stimuli for adaptation, resulting in similar improvements. From a practical perspective, different scheduling strategies could be used interchangeably depending on the available context (e.g., time constraints, low-fatigue tasks, and adherence).

This study has some limitations. No control group was included, making it difficult to rule out learning effects. Only men were investigated, which limits the generalisation of findings to women. Except sports club participation, no other variables were collected that could influence balance performance. The relatively long recovery (≥48 h) between sessions may have been sufficient to allow similar adaptation which minimized training-related differences between groups.

## Conclusions

The present work provides novel insights into youth by showing that both massed and distributed practice led to similar improvements in balance, indicating that both BT distribution strategies can be used in adolescents depending on resources and constraints for practice.

## Conflict of interest

The authors declare that they have no conflict of interest.
